# Machine learning models combining computed tomography semantic features and selected clinical variables for accurate prediction of the pathological grade of bladder cancer

**DOI:** 10.3389/fonc.2023.1166245

**Published:** 2023-05-08

**Authors:** Zhikang Deng, Wentao Dong, Situ Xiong, Di Jin, Hongzhang Zhou, Ling Zhang, LiHan Xie, Yaohong Deng, Rong Xu, Bing Fan

**Affiliations:** ^1^ Medical College of Nanchang University, Nanchang University, Nanchang, China; ^2^ Department of Nuclear Medicine, Jiangxi Provincial People’s Hospital, The First Affiliated Hospital of Nanchang Medical College, Nanchang, China; ^3^ Department of Radiology, Jiangxi Provincial People’s Hospital, The First Affiliated Hospital of Nanchang Medical College, Nanchang, China; ^4^ Department of Urology, The First Affiliated Hospital of Nanchang University, Nanchang, China; ^5^ Department of Research & Development, Yizhun Medical AI Co. Ltd., Beijing, China

**Keywords:** bladder cancer, pathological grade, combined radiomics nomogram, textural features, non-enhanced computed tomography

## Abstract

**Objective:**

The purpose of this research was to develop a radiomics model that combines several clinical features for preoperative prediction of the pathological grade of bladder cancer (BCa) using non-enhanced computed tomography (NE-CT) scanning images.

**Materials and methods:**

The computed tomography (CT), clinical, and pathological data of 105 BCa patients attending our hospital between January 2017 and August 2022 were retrospectively evaluated. The study cohort comprised 44 low-grade BCa and 61 high-grade BCa patients. The subjects were randomly divided into training (*n* = 73) and validation (*n* = 32) cohorts at a ratio of 7:3. Radiomic features were extracted from NE-CT images. A total of 15 representative features were screened using the least absolute shrinkage and selection operator (LASSO) algorithm. Based on these characteristics, six models for predicting BCa pathological grade, including support vector machine (SVM), k-nearest neighbor (KNN), gradient boosting decision tree (GBDT), logical regression (LR), random forest (RF), and extreme gradient boosting (XGBOOST) were constructed. The model combining radiomics score and clinical factors was further constructed. The predictive performance of the models was evaluated based on the area under the receiver operating characteristic (ROC) curve, DeLong test, and decision curve analysis (DCA).

**Results:**

The selected clinical factors for the model included age and tumor size. LASSO regression analysis identified 15 features most linked to BCa grade, which were included in the machine learning model. The SVM analysis revealed that the highest AUC of the model was 0.842. A nomogram combining the radiomics signature and selected clinical variables showed accurate prediction of the pathological grade of BCa preoperatively. The AUC of the training cohort was 0.919, whereas that of the validation cohort was 0.854. The clinical value of the combined radiomics nomogram was validated using calibration curve and DCA.

**Conclusion:**

Machine learning models combining CT semantic features and the selected clinical variables can accurately predict the pathological grade of BCa, offering a non-invasive and accurate approach for predicting the pathological grade of BCa preoperatively.

## Introduction

Bladder cancer (BCa) is a malignant tumor of the bladder mucosa. It is the 11^th^ most prevalent malignant tumor in the world (1) and the most common malignancy of the urinary system (2). In 2022, an estimated 81,180 new BCa cases were reported in the United States, resulting in 17,100 deaths in the year (3). The pathological grade is a critical parameter in determining the sensitivity to treatment and prognosis (4). BCa is classified into low grade and high grade based on the morphological differences in the nucleus and the mitotic image of tumor cells (5). Low-grade BCa progresses slowly and rarely threatens the life of patients. It requires initial endoscopic treatment and monitoring. However, some cases of low-grade BCa can be invasive (6, 7). High-grade BCa has a high malignant potential associated with rapid progression and high mortality (8, 9). Given the high risk of high-grade BCa progression, full dose Bacillus Calmette-Guerin (BCG) vaccine is recommended for 1 to 3 years in the bladder, and radical cystectomy may also be considered (10). The risk of low-grade and high-grade BCa progression has been reported to be approximately 2.6 and 13.7%, respectively (11). In general, the overall survival time of patients with high-grade BCa is poorer compared with that of patients with low-grade BCa (12). The pathological grade of BCa is mainly obtained through pathological examination. However, pathological sampling is inadequate due to the physical nature of biopsy and transurethral excised specimens. Therefore, other methods are needed to accurately distinguish between low- and high-grade Bca preoperatively, which can significantly shorten the diagnosis and treatment pathway.

Computed tomography (CT) examination is one method for examining the bladder for tumors. The difference between CT and non-enhanced computed tomography (NE-CT) is mainly the use or absence of contrast media. CT uses contrast media to provide higher resolution, sharper images, accurate lesion localization, and more comprehensive diagnostic information. However, CT also carries the risk of adverse reactions such as hypersensitivity to contrast media and renal impairment, and is costly. In contrast, the advantages of NE-CT include lower cost, no need to inject contrast agent and does not cause uncomfortable reactions due to allergy to contrast agent. However, because no contrast agent is used, NE-CT may lead to poor image quality, difficulty distinguishing lesions from normal tissue, and inability to detect certain lesions in some cases. Zhang et al. (13) investigated the feasibility of using unenhanced and enhanced images to differentiate low-grade and high-grade urothelial cancer. However, based on diagnostic performance, quantitative CT texture analysis revealed that it was impossible to differentiate the pathological grade of urothelial carcinoma of the bladder and upper tract urothelial carcinoma. Magnetic resonance imaging (MRI) technology has a multi-parameter, multi-angle, and multi-azimuth imaging and overcomes the shortcomings of CT. Zhang et al. (14) demonstrated that textural features from apparent diffusion coefficient (ADC) and diffusion-weighted image (DWI) maps can reflect the discrepancy between low-grade and high-grade BCa. Wang et al. (15) revealed that the MRI-based multiparametric radiomics method could be used as a non-invasive imaging tool for evaluating the pathological grade of BCa preoperatively. Zheng et al. (16) showed that the multi-parameter MRI (mpMRI) radiomics approach could predict the pathological grade of BCa preoperatively. Nevertheless, CT is more common than MRI in clinical practice, especially in third-world countries. To date, no model has been reported for predicting the pathological grade of BCa using NE-CT. In the present study, a model for predicting the pathological grade of BCa was developed using NE-CT data.

Tumor heterogeneity cannot be reliably evaluated visually. Radiomics is a new method in radiology that extracts and applies data in a clinical decision support system to promote the prognosis, prediction, and accuracy of diagnosis (17, 18). Radiomics is a non-invasive method that evaluates tumors and their microenvironment and monitors tumor characteristics. Several studies have demonstrated that this new method for predicting the pathological grade of BCa can be used as an alternative for MRI and CT qualitative analysis and can identify information that is invisible to the human eye (14–17). Therefore, this research developed a nomogram that combines several radiomic features to predict the pathological grade of BCa.

## Materials and methods

### Study setting and participants

One hundred and seventy-seven BCa patients attended treatment at the Nanchang Medical College Hospital between January 2017 and August 2022. The data had been reviewed and formally accepted. The protocol for this study was approved by the Ethics Committee of the First Affiliated Hospital of Nanchang Medical College. Informed consent was not required. All relevant codes and regulations for this study are applied worldwide. The inclusion criteria for the patients were as follows: (1) There were clear pathological grading data of BCa, (2) the NE-CT image was complete for lesion evaluation, and (3) CT examination was performed 14–30 days before operation. The exclusion criteria for the patients were as follows: (1) The CT imaging quality was not ideal, mainly due to the presence of significant artifacts; (2) patients undergone immunotherapy or chemotherapy before CT examination; (3) the size of the lesion was less than 5 mm or only showed thickening of the bladder wall; (4) missing or incomplete clinical and pathological data. In the end, 105 patients were included in the study. The subjects were randomly divided into training (*n* = 73) and validation (*n* = 32) cohorts at a ratio of 7:3. The study cohort comprised 44 low-grade and 61 high-grade BCa patients.

### Examination methods

Siemens SOMATOM Definition dual source CT was used for routine plain scanning of the abdomen or pelvis. Scanning parameters were as follows: tube voltage of 120 Kv, tube current of 150 As, scanning layer thickness of 5 mm, a reconstruction layer thickness of 1 mm, and layer spacing of 1 mm.

### Region of interest segmentation

**Figure 1:**
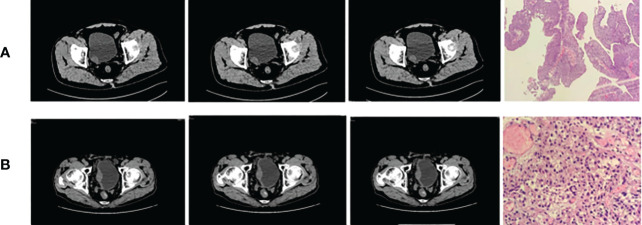
Bladder cancer **(A)** on the right wall (low grade) and **(B)** on the right rear wall (high grade).

**Figure 2:**
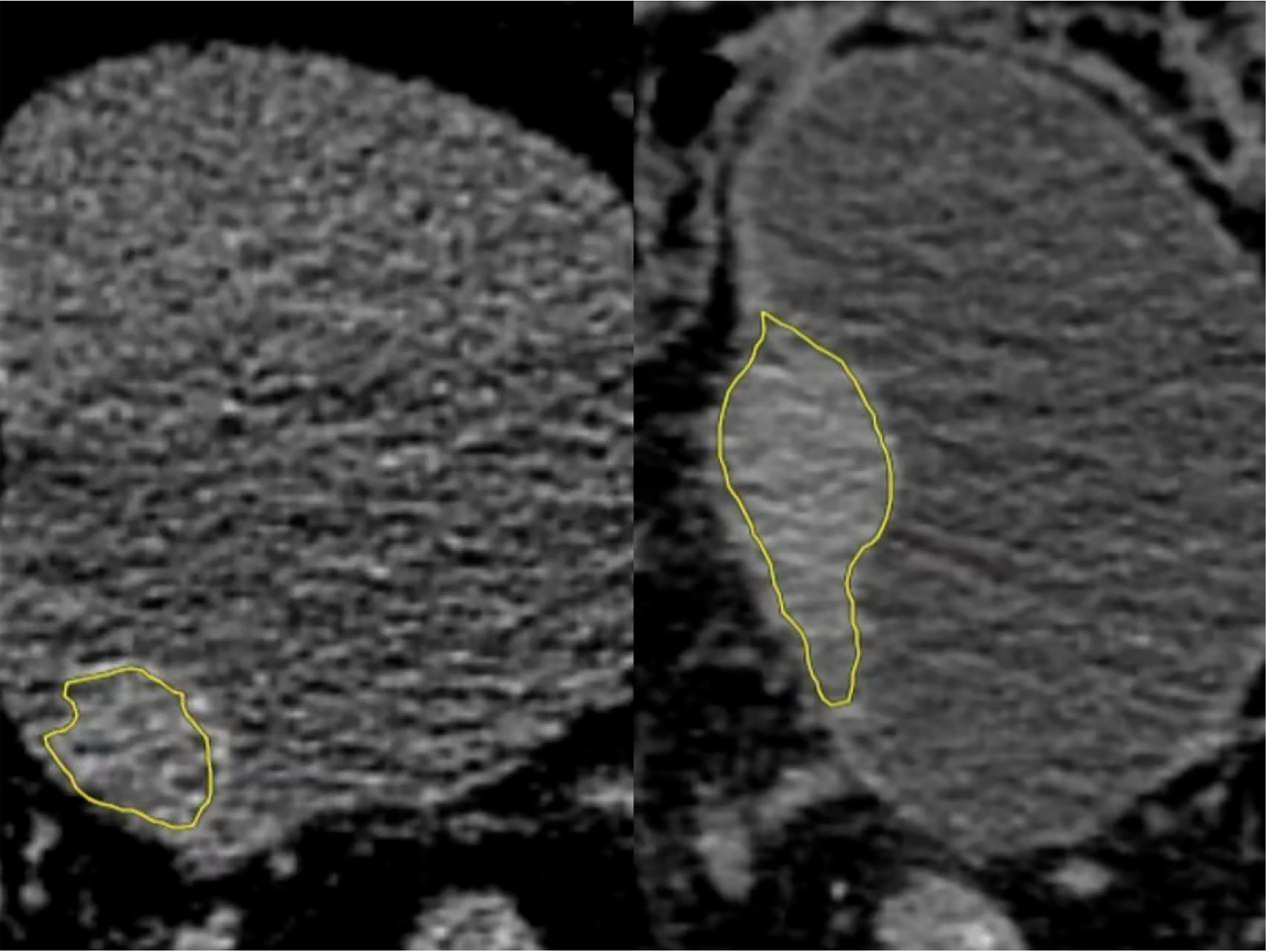
Low-grade (left) and high-grade (right) regions of interest (ROIs). They were manually outlined in all layers of bladder cancer on the NE-CT images using image processing software (DARWIN intelligent scientific research platform), which were merged into a three-dimensional ROI diagram (yellow).

The CT image was segmented by an experienced radiologist (Reader A, with 5 years of experience in urogenital imaging). Areas of interest were outlined using DARWIN intelligent scientific research platform (19). One senior radiologist (Reader B, with 15 years of experience in urogenital imaging) reviewed all the region of interest (ROI) segmented by Reader A. If ROI was diverse, the senior radiologist determined the lesion boundary (**Figures 1**, **2**).

### Feature extraction and selection in radiomics

**Figure 3:**
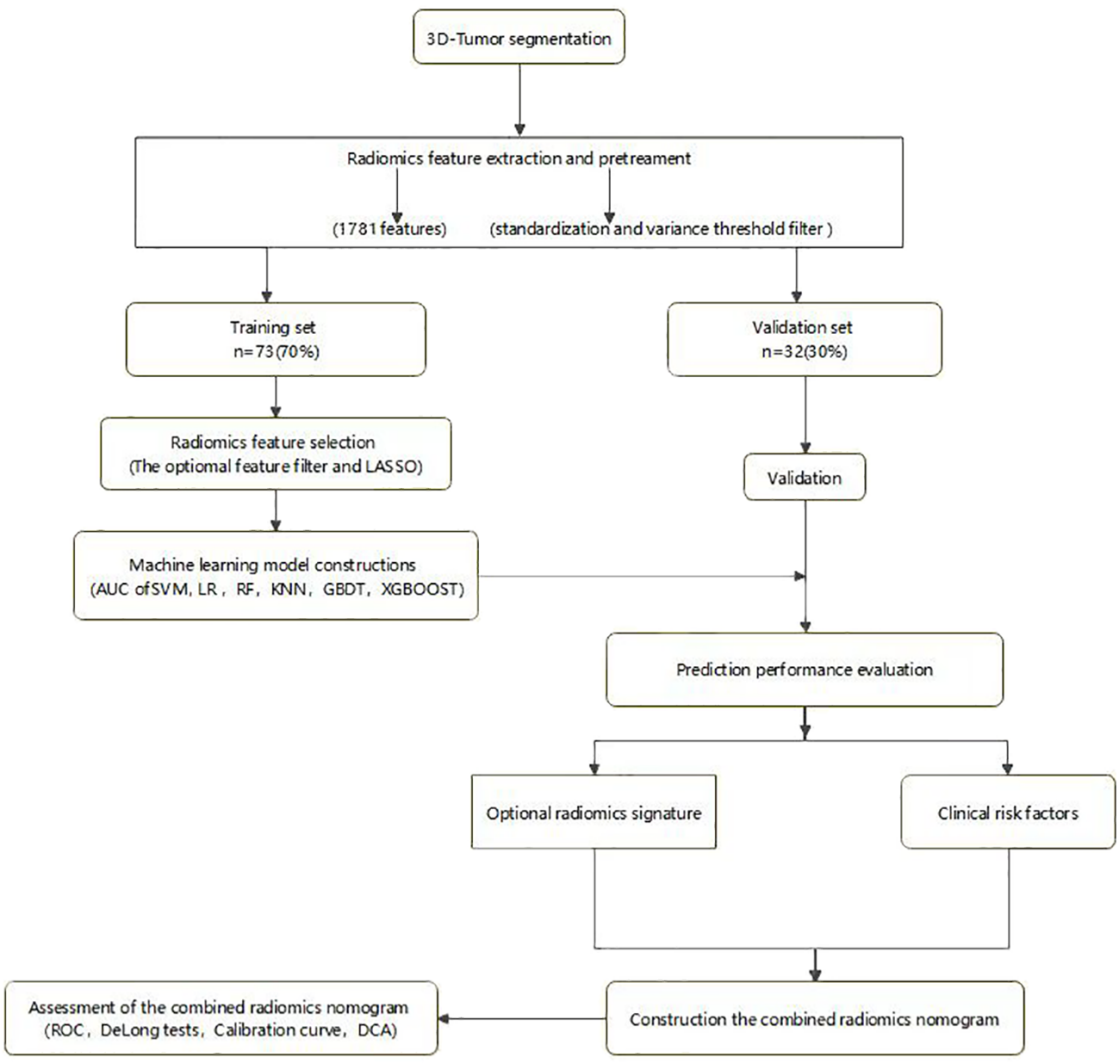
Radiomics workflow.

**Figure 4:**
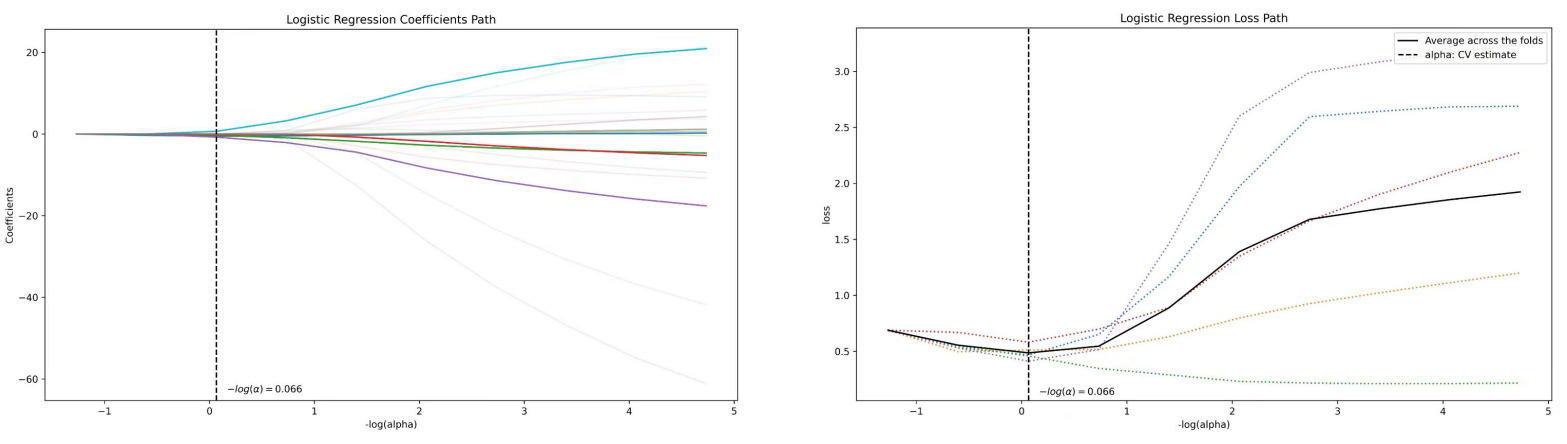
Feature selection using the LASSO algorithm (left, LASSO path; right, MSE path).

**Figure 5:**
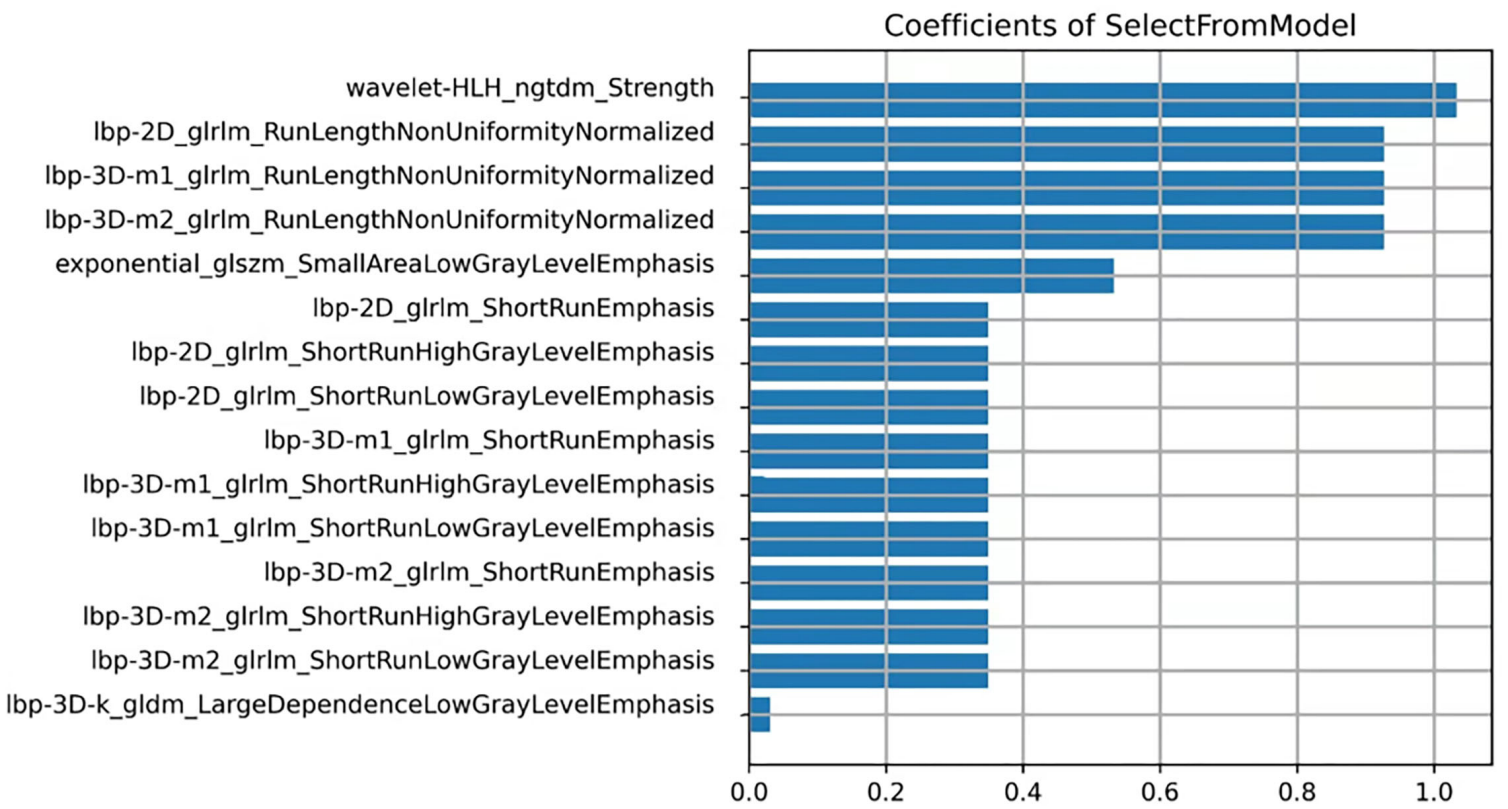
The final 15 features selected (15 textures).

A total of 1,781 radiomic features were using DARWIN intelligent scientific research platform (**Figure 3**). Extracted features included 14 shape features, 18 first-order features, 24 GLCM features, 14 GLDM features, 16 GLRLM features, 16 GLSZM features, 5 NGTDM features, and 18 groups of transformed features, which had 93 features. The platform extracted a group of LoG features, which sigma was 3.0 besides default transformed features in pyradiomics. The Force 2D extraction (A default parameter in the Feature Class Level) was configured on the platform. For classification, the attributes of each dimension were linearly stretched to an interval through standardization and variance threshold filter. The data were preprocessed to obtain an appropriate model. The computer-generated data set was randomly allocated, with 70% of the data set assigned to the training cohort (30 low-grade groups and 43 high-grade groups) and 30% allocated to the validation cohort (13 low-grade groups and 19 high-grade groups). In the training of classifier, we added feature selection. Linear correlation between each feature and the category label was evaluated through the optimal feature filter, and the 45 most relevant features from the 1,781 features were selected. The LASSO algorithm was used to select the most relevant feature from the 45 features (20) (**Figure 4**). Finally, we selected a total of 15 most relevant features for the pathological grade of BCa (**Figure 5**).

### Radiomics signature construction

The optimal subset was selected by decreasing the proportion. Six machine learning models, namely, support vector machine (SVM), k-nearest neighbor (KNN), gradient boosting decision tree (GBDT), logical regression (LR), random forest (RF), and extreme gradient boosting (XGBOOST), were constructed. The predictive performance of the six models was further tested in the independent validation cohort using the same threshold data set in the training cohort. The 10-fold cross-validation method was used to verify the accuracy of the model. Receiver operating characteristic (ROC) and area under the curve (AUC) were used to evaluate the performance of the six models, and the accuracy, sensitivity, and specificity of the models were determined. The best model was then selected from six machine-learning models.

### Statistical analysis

The statistical analysis was performed using SPSS software version 25.0 and R statistical tool (Version 3.4.4). Wilcoxon rank-sum test (skewed distribution) and t-test (normal distribution) were used to compare probability scores of low-grade and high-grade BCa. The chi-square test was used to compare data between the two groups. The predictive performance of the model was evaluated by calculating accuracy, sensitivity, specificity, and AUC value.

## Results

### Patient characteristics

In this study, 105 patients were randomly assigned to the training cohort (*n* = 73) and the validation cohort (*n* = 32). All patients had definite pathological findings, including 44 cases of low-grade BCa and 61 cases of high-grade BCa. There were no significant differences between patients in the training cohort and those in the validation cohort in terms of gender, number, number_multiple, smoothing, hematuria, and neutrophil to lymphocyte ratio (NLR). However, in low-grade and high-grade BCa patients, age and tumor size were significantly different (**Table 1**). Univariate logistic regression (LR) analysis was performed to determine the effect of each variable on the pathological grade of BCa. The results showed that age (*P* < 0.01), size (*P* < 0.01) and NLR (*P* < 0.05) were significantly correlated with the pathological grade of BCa, while other variables were not. Multivariate LR analysis was conducted based on the univariate LR analysis. It was observed that only age, *OR* = 1.08, 95% CI [1.02,1.15], *P* < 0.05, and size, adjusted *OR* = 1.75, 95% CI [1.07; 2.84], *P* < 0.05, were significantly correlated with the pathological grade of BCa (**Table 2**).

**Table 1:** Clinical and imaging characteristics of the training and validation cohorts.

	Training cohort (n = 73)		P-value	Validation cohort (n = 32)	P*-value
	Low-grade	High-grade			
Age	61.47 ± 11.34	68.79 ± 9.87	0.005	72.09 ± 9.035	0.005 0.33
Sex	2 (2.74%)		0.386		0.338
female	28 (38.36%)	7 (9.59%)		7 (21.88%)	0.231
male	1.75 (1.25, 2.76)	36 (49.32%)		25 (78.12%)	0.349
Size	1.97 ± 1.65	3.09 (1.84, 3.95)	0.002	2.59 (1.76,4.47)	0.178
Number	19 (26.03%)	1.72 ± 1.75	0.548	1.50 ± 1.39	
Number_Multiple	11 (15.07%)	31 (42.47%)	0.428	26 (81.25%)	0.016
NO		12 (16.44%)		6 (18.75%)	
YES	24 (32.88%)				0.970
Smoothing	6 (8.22%)	33 (45.21%)		1 (3.10%)	
No		10 (13.70%)	0.741	31 (96.90%)	0.326
Yes	5 (6.85%)				0.302
Hematuria	25 (34.25%)	13 (17.81%)		8 (25.00%)	
No	2.43 (1.71, 3.15)	30 (41.10%)	0.186	24 (75.00%)	
Yes	-0.45 ± 0.63	2.73 (2.00, 5.00)		2.69 (2.12, 4.40)	
NLR		0.73 ± 0.58	0.050	0.39 ± 0.55	
Rad-score			< 0.001		

NLR, neutrophil to lymphocyte ratio.

P < 0.05: significant difference between the low-grade and high-grade groups in the training cohort.

P* represents < 0.05: significant difference between training and validation cohorts.

**Table 2:** Logistic regression analysis for predicting the pathological grade of bladder cancer.

Variable	Univariate regression			Multivariate regression		
	Odds ratio	(95% CI)	P-value	Odds ratio	(95% CI)	P-value
Age	1.07	[1.02;1.12]	0.008	1.08	[1.02;1.15]	0.013
Sex	0.37	[0.07;1.91]	0.233	NA	NA	NA
Size	1.94	[1.23;3.05]	0.004	1.75	[1.07;2.84]	0.025
Number	0.92	[0.70;1.21]	0.544	NA	NA	NA
Number_multiple	0.67	[0.25;1.81]	0.429	NA	NA	NA
Smoothing	1.21	[0.39;3.79]	0.741	NA	NA	NA
Hematuria	0.46	[0.14;1.47]	0.191	NA	NA	NA
NLR	1.42	[1.04;1.93]	0.028	1.41	[0.99;2.01]	0.061

NA, Not Available.

### Predictive performance of machine learning models

**Figure 6:**
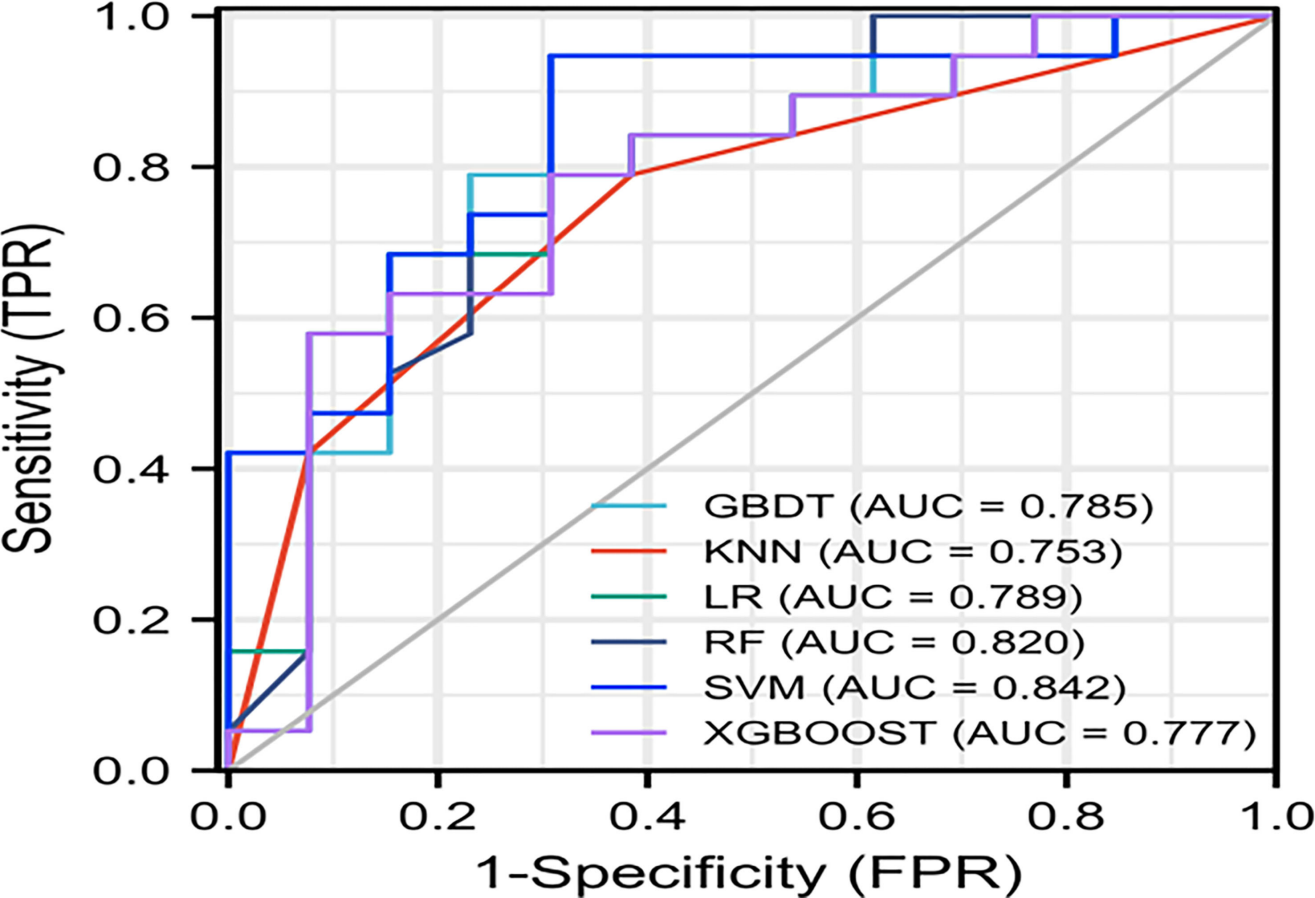
ROC curves of the six machine learning models in the validation cohort.

**Figure 7:**
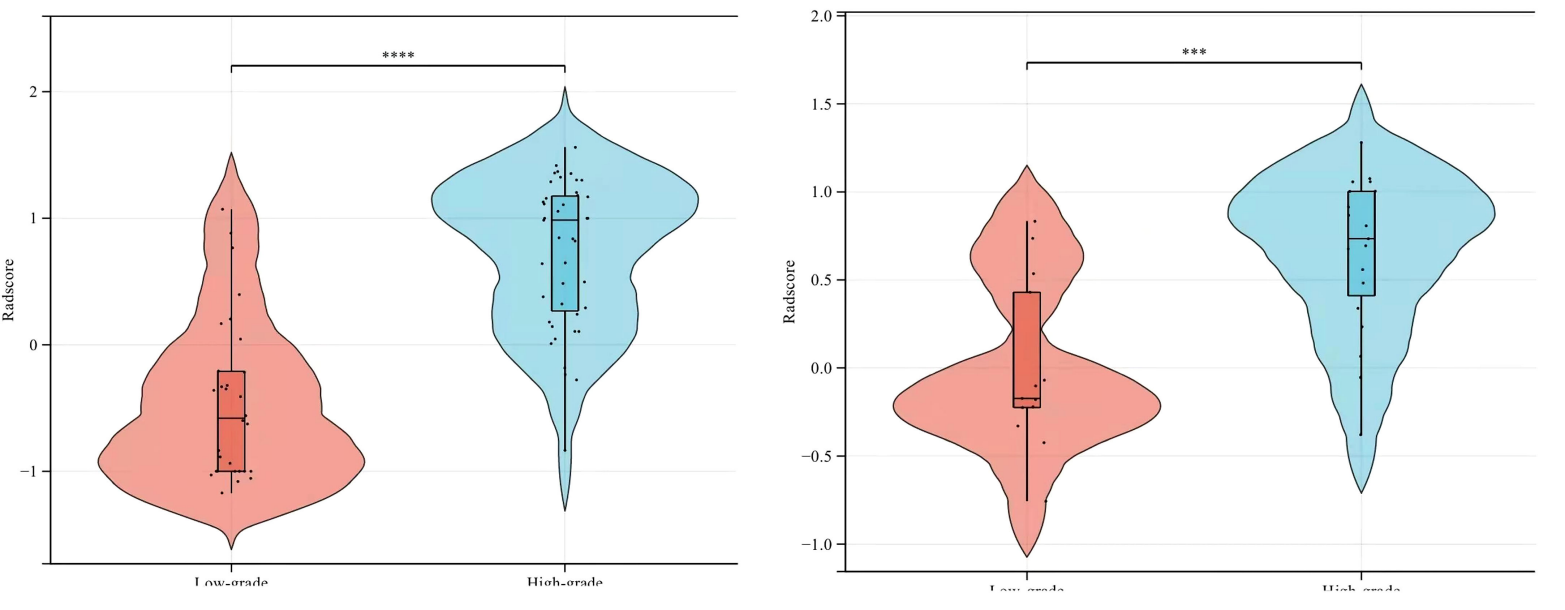
Comparison of radiomics score between low-grade and high-grade bladder cancer in the training (left) and validation (right) cohorts.

The AUC values of SVM, KNN, GBDT, LR, RF, and XGBOOST in the training cohort were 0.909, 0.895, 1.000, 0.908, 1.000, and 1.000, respectively. The AUC values of SVM, KNN, GBDT, LR, RF, and XGBOOST in the validation cohort were 0.842, 0.753, 0.785, 0.789, 0.820, and 0.777, respectively (**Table 3**). The ROC curves of the six machine-learning models are shown in **Figure 6**. The SVM was the best radiomics model in the validation cohort, with the most effective performance. The accuracy, sensitivity, specificity, and AUC values were 0.844, 0.947, 0.692, and 0.842, 95% CI: [0.699, 0.985], respectively. Cross-validation was performed in the training cohort to obtain a series of optimal hyperparameters. The sample difference between low-grade and high-grade radiomics score during training and testing was significant (**Figure 7**). This demonstrated that the radiomics features were related to the pathological grade of BCa.

**Table 3:** Predictive performance of six machine learning models in the training and validation cohorts.

	Training cohort				Validation cohort			
	ACC	SEN	SPE	AUC	ACC	SEN	SPE	AUC
SVM	0.849	0.93	0.767	0.909	0.843	0.947	0.692	0.842
LR	0.876	0.884	0.867	0.908	0.781	0.684	0.846	0.789
RF	1	1	1	1	0.843	0.947	0.692	0.82
GBDT	0.986	1	1	1	0.75	0.789	0.769	0.785
KNN	0.835	0.837	0.8	0.895	0.687	0.789	0.615	0.753
XGBOOST	0.986	1	1	1	0.718	0.579	0.923	0.777

### Combined radiomics model

**Figure 8:**
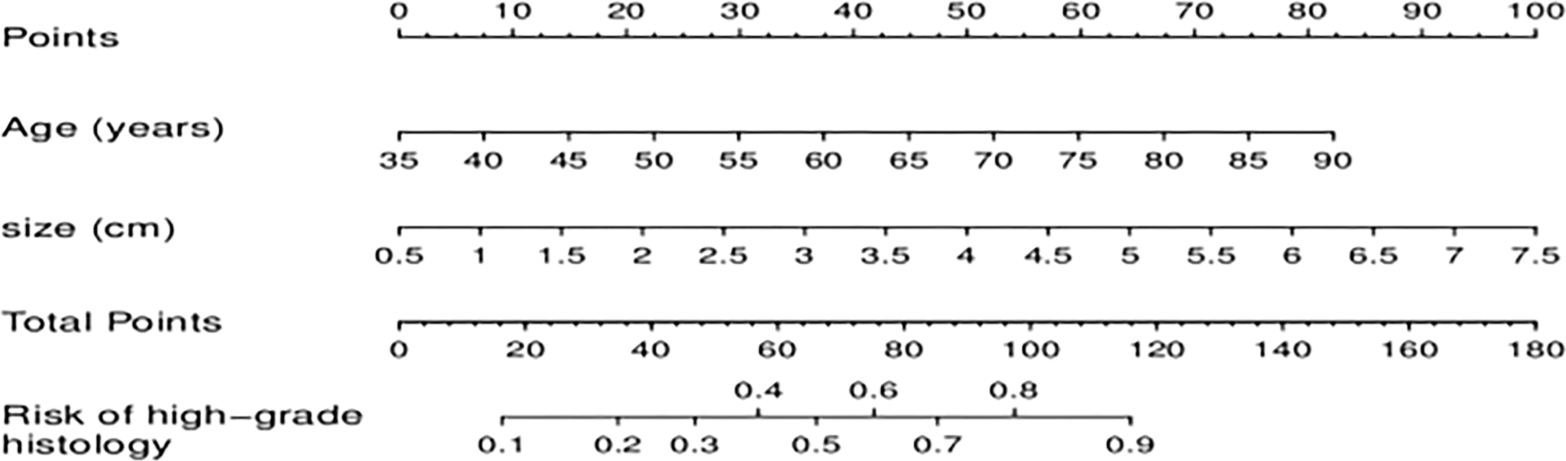
A combined radiomics nomogram for predicting pathological grade of bladder cancer.

Based on the clinical variables, multivariate LR analysis revealed that only age and tumor size were independent predictive variables of low-grade and high-grade BCa. Subsequently, the clinical model was established and confirmed in the validation cohort according to the above predictive variables. The AUC values of the training and validation cohorts were 0.760 and 0.753, respectively. The AUC value of the radiomics model was 0.909 in the training cohort, whereas that in the validation cohort was 0.842. To establish a clinically applicable and more accurate model to predict the pathological grade of BCa, the LR algorithm was used to construct a nomogram that combined age, tumor size, and NE-CT radiomics features (**Figure 8**).

**Figure 9:**
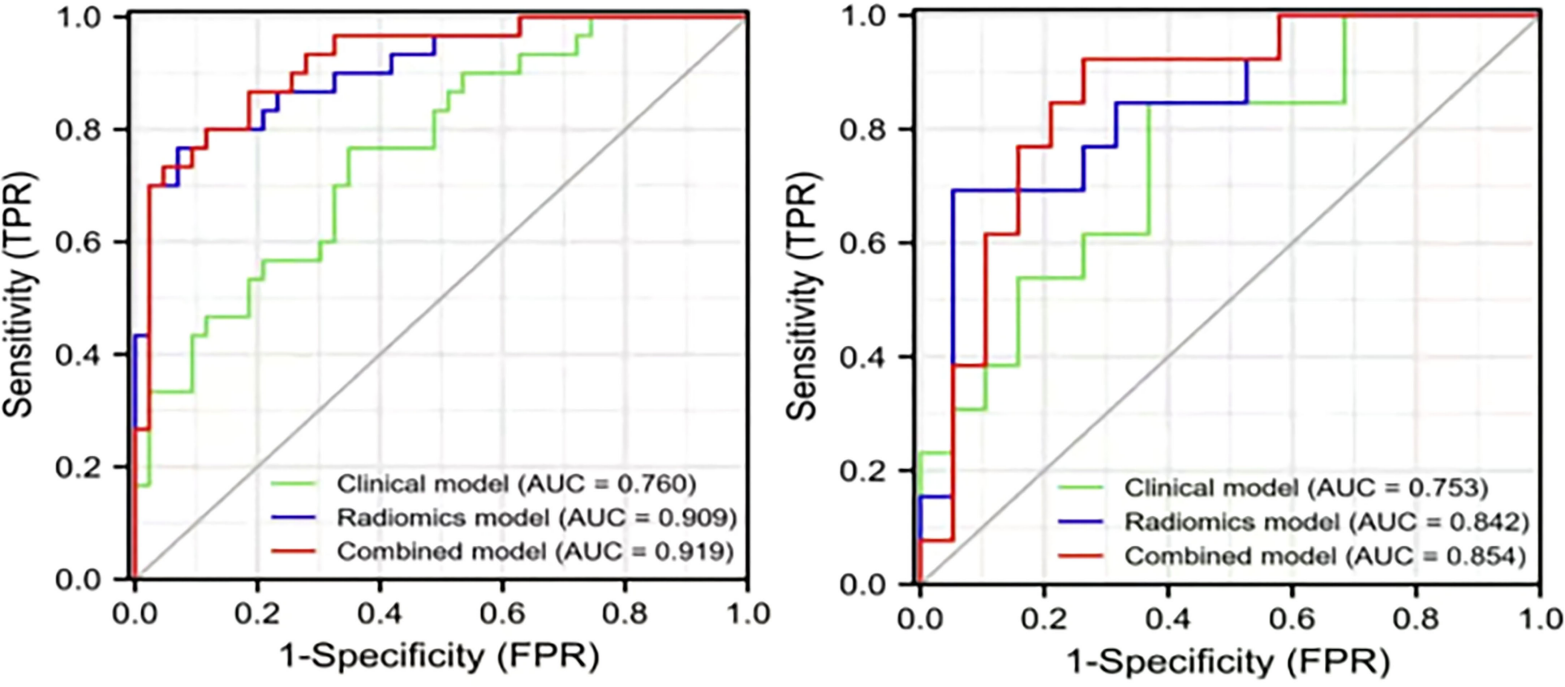
The AUC values for the clinical, radiomics, and combined models used to distinguish between low-grade and high-grade bladder cancer. Training cohort (left); validation cohort (right).

**Figure 10:**
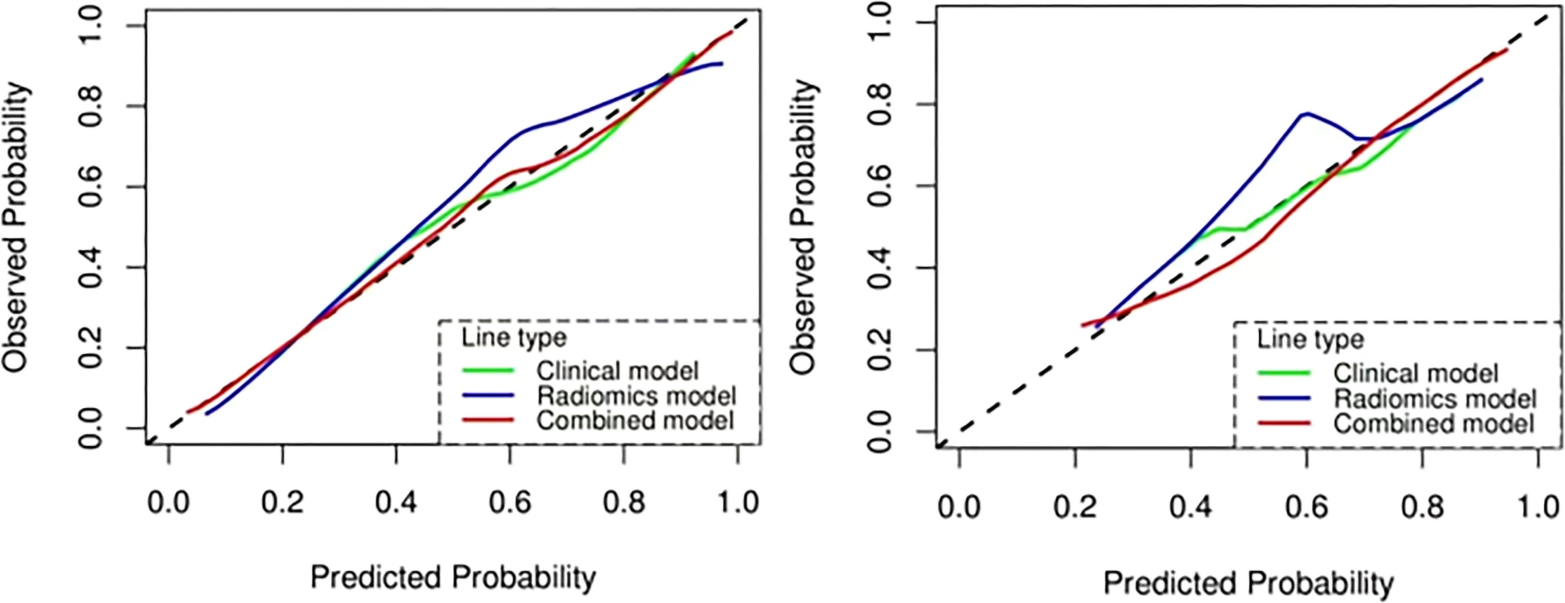
The calibration curve analysis of the clinical, radiomics, and combined models. Training cohort (left); validation cohort (right).

**Figure 11:**
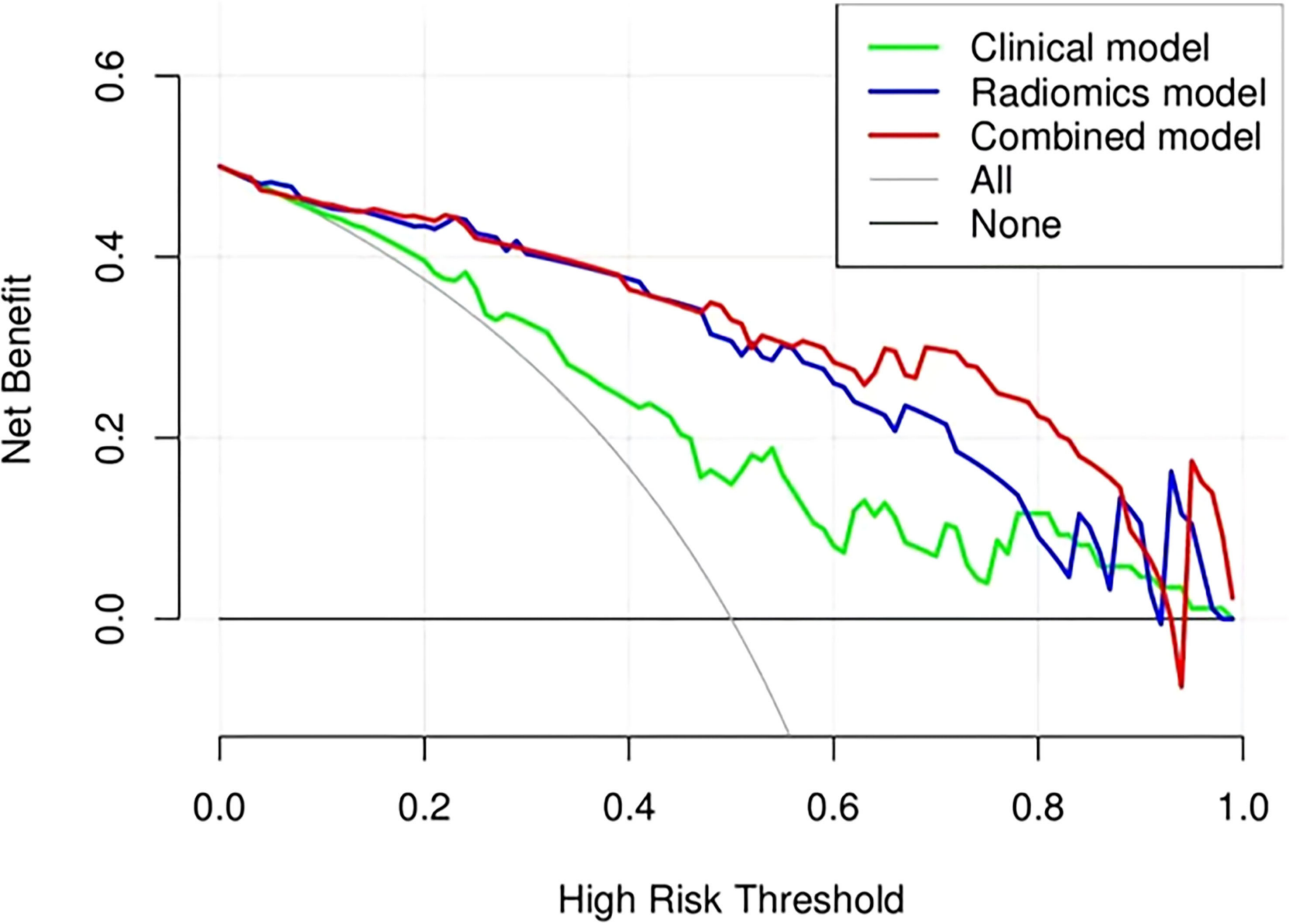
Decision curve analysis (DCA) of the clinical, radiomics, and combined models in the combined training and validation cohorts.

The ROC analysis confirmed the identification effect of the nomogram that combines radiomics features with an AUC value of 0.919 for the training cohort and 0.854 for the validation cohort (**Figure 9**). AUC values of radiomics and clinical models were lower than those of the training and validation cohorts. The *P*-value for the difference between the clinical and combined models in the validation cohort based on the DeLong test was higher than 0.05. The stratification accuracy of the nomogram that combined radiomics features was significantly higher than the radiomics and clinical models. The difference in AUC values between the two models was statistically significant (*p*<0.05), otherwise not (**Table 4**). The calibration curves of the three models, namely, clinical, radiomics, and combined models, demonstrated an outstanding consistency in the actual and predicted pathology grade of BCa (**Figure 10**). DCA showed that the nomogram that combined radiomics features had maximum clinical practicability, indicating that it was a reliable clinical tool for predicting the pathological grade of BCa (**Figure 11**).

**Table 4:** Comparison of the stratification prediction accuracy of the clinical, radiomics, and combined models.

Group	Model 1	Model 2	P-value
Training			
	Clinical	Radiomics	0.016
	Clinical	Combined	0.002
	Radiomics	Combined	0.520
Validation			
	Clinical	Radiomics	0.395
	Clinical	Combined	0.200
	Radiomics	Combined	0.761

## Discussion

Even though cystoscopy biopsy plays an important role in predicting BCa prognosis, it may sometimes underestimate pathological grade (21). The proposed radiomics model can extract information from the whole tumor, enhancing accurate diagnosis. Zhang et al. (22) predicted the pathological grade of BCa using the LR machine learning model. The AUC value of the model in the training cohort was 0.950 and 0.860 for the validation cohort. However, clinical indicators were not included in the LR model. Feng et al. (23) used a nomogram that combines radiomics features to predict the pathological grade of BCa. The AUC value of the ADC1700 model for differentiating low-grade BCa from high-grade BCa was 0.920 in the training cohort and 0.745 in the testing cohort. However, only the LR model was used to explore the nomogram that combines radiomics features without analyzing the possibility of other models. Therefore, based on the six machine learning models, this study selected the optimal model to analyze the clinical features of BCa combined with radiomics and constructed a nomogram that combines radiomics features. The radiomics model incorporated clinical features, which further enhanced the classification accuracy. The radiomics features can only reflect the information displayed in NE-CT images, whereas the clinical features provide other indicators for identifying disease risks. In our study, we combined radiomics and clinical factors to obtain the best performance.

The clinical features selected in this study were age and tumor size, which were obtained using multivariate LR analysis. There was no significant difference in other features in predicting the pathological grade of BCa (**Table 2**). The reason may be because the proportion of poorly differentiated tumor cells gradually decreases, and the proportion of highly differentiated tumor cells gradually increases with age. The older the patient, the higher the pathological grade and the higher the risk of cancer invasiveness. The accumulation of carcinogenic factors occurs over time and space. Low-grade BCa tumors are usually small, with regular bladder contour, uniform density, and clear fat space around the bladder. In contrast, high-grade BCa is a large mass with irregular bladder contour, uneven internal density or signal, and partial narrowing or disappearance of fat space around the bladder.

This research used a NE-CT-based nomogram that combines radiomics features to differentiate between low-grade and high-grade BCa. The constructed nomogram based on clinical independent factors and radscore was non-invasive, convenient, and rapid. CT radiomics image analysis can objectively evaluate the heterogeneity of lesions and organs, revealing information on the tissue microenvironment more accurately than subjective visual interpretation. Tumors can show heterogeneity in many aspects, such as cellular, genetic, and phenotypic levels (24). Cancers are challenging to categorize using subjective and traditional approaches due to their diverse tumor heterogeneity. Radiomic imaging techniques are presently employed to anticipate the cancerousness of different solid tumors and the pathological degree, extent of invasion, or response to therapy of malignant tumors.

The 3D-ROI was used for feature extraction. A total of 15 radiomics features were selected (**Figure 5**). The SVM was used to obtain the best predictive radiomics model. An SVM is an effective, powerful, and robust machine learning classifier used primarily in radiology (25, 26). In this study, SVM had faster training and classification speed than RF, KNN, LR, GBDT, and XGBOOST, because it was most suitable for high-dimensional features (27). The SVM was superior to the RF, KNN, LR, GBDT, and XGBOOST, with the highest AUC value (0.842) in the relevant validation cohort (**Table 3**, **Figure 6**). A nomogram was constructed that combined relevant clinical features and radiomics scores. The AUC value based on the training cohort was 0.919 and 0.854 for the validation cohort, higher than those of the radiomics and clinical models (**Figure 9**). The stratification accuracy of the nomogram that combined radiomics features was superior to that of the models relying on radiomics or clinical risk factors alone. This result indicates that the nomogram that combined radiomics features based on limited sample information can be an optimal model for learning ability and model complexity. This model can solve nonlinearity, high dimensions, and small sample sizes (28). The combined model has been widely used in medical practice in recent years (29). Risk scores were used to represent the risk factors for predicting the treatment effect of different diseases. The combined model is simple and easier to understand than the radiomics and clinical models.

The constructed nomogram that combines radiomics features in this study can accurately predict BCa with different pathological grades based on clinical and radiomics features of NE-CT. In this research, 15 radiomics features, including second-order and higher-order texture features, were selected using the LASSO algorithm. NGTDM was the most accurate in distinguishing low-grade and high-grade BCa. Compared with other image features, NGTDM features can better reflect changes in the internal structure of the organization. It describes the difference in gray level between pixels in digital images and indicates the change in internal structure of different regions. For patients with bladder cancer, NGTDM features can effectively characterize differences between cancer cells and normal tissues. Therefore, it has higher accuracy and sensitivity for the pathological grade of bladder cancer.

NGTDM characteristics include roughness, busyness, strength, contrast, and complexity. Several studies have verified its accuracy. According to the study conducted by Gökçen Ç et al. (30), NGTDM was a technique that could be used to express the spatial diversity of pixel intensity. Song H et al. (31) reported that these characteristics can describe the local tumor texture based on differences between each voxel and the adjacent voxels in the adjacent image plane. A study by Liu J et al. (32) revealed that higher value of strength_NGTDM_ indicated an image with slower change in intensity but larger coarse differences in gray level intensities. However, this study did not combine most pathological and physiological radiomics features. Although the model combined radiomics features, information on the role and potential biological mechanism of these features in the model is scanty. Therefore, further studies are needed to explore its potential value.

This study had limitations. First, being a retrospective study, selection bias cannot be ruled out. Second, the sample size used in this research was relatively small. Therefore, studies utilizing large sample sizes are needed to validate our findings. Finally, this study was a single-center study. The multi-center collaborative study is still needed (33). To enhance the dependability of the nomogram developed in this investigation, it is necessary to validate it with data obtained from medical institutions in other geographical areas.

In conclusion, the SVM model based on NE-CT data radiomics feature extraction has excellent prediction accuracy and reliability and could be used for predicting the pathological grade of BCa preoperatively. The combined radiomics nomogram further enhances the pathological grade stratification accuracy of BCa. According to the different pathological grades of BCa, this model can be used to guide on the surgical methods. In the future, radiomics research is expected to become more advanced. By utilizing machine learning and deep learning techniques, imaging features can be automatically recognized and evaluated to provide clinical guidance, thereby streamlining the workflow of healthcare professionals and enhancing diagnostic efficiency (34). There are some problems with the quality and comparability of radiomics data due to technical differences and operational codes between different research institutions and laboratories. Hence, promoting standardization and normalization of radiomics will be a crucial area of advancement, as it can enhance the consistency and reliability of data, leading to better support for clinical practice (35).

## Data availability statement

The original contributions presented in the study are included in the article/supplementary material. Further inquiries can be directed to the corresponding authors.

## Ethics statement

The studies involving human participants were reviewed and approved by Medical Ethics Committee of Jiangxi Provincial People’s Hospital. Written informed consent for participation was not required for this study in accordance with the national legislation and the institutional requirements. Written informed consent was not obtained from the individual(s) for the publication of any potentially identifiable images or data included in this article.

## Author contributions

The authors made the following contributions: ZD and RX made the conception for this research. Data collection and analysis were performed by ZD, WD, DJ, BF, YD and SX. ZD, WD,LX and HZ analyzed the data and drafted the article. RX, WD, SX and LZ reviewed/edited the manuscript. All the authors critically revised the article for important intellectual content. All authors contributed to the article and approved the submitted version.

## References

[1] Teoh JY Huang J Ko WY Lok V Choi P Ng CF. Global trends of bladder cancer incidence and mortality, and their associations with tobacco use and gross domestic product per capita. Eur Urol (2020) 78(6):893–906. doi: 10.1016/j.eururo.2020.09.006 32972792

[2] Crocetto F Buonerba C Caputo V Ferro M Persico F Trama F. Urologic malignancies: advances in the analysis and interpretation of clinical findings. Future Sci OA (2021) 7(4):FSO674. doi: 10.2144/fsoa-2020-0210 33815820 PMC8015670

[3] Sung H Ferlay J Siegel RL Laversanne M Soerjomataram I Jemal A. Global cancer statistics 2020: GLOBOCAN estimates of incidence and mortality worldwide for 36 cancers in 185 countries. CA Cancer J Clin (2021) 71(3):209–49. doi: 10.3322/caac.21660 33538338

[4] Varma M Delahunt B van der Kwast T. Grading noninvasive bladder cancer: world health organisation 1973 or 2004 may be the wrong question. Eur Urol (2019) 76(4):413–5. doi: 10.1016/j.eururo.2019.05.001 31080126

[5] Netto GJ Amin MB Berney DM Compérat EM Gill AJ Hartmann A. The 2022 world health organization classification of tumors of the urinary system and Male genital organs-part b: prostate and urinary tract tumors. Eur Urol (2022) 82(5):469–82. doi: 10.1016/j.eururo.2022.07.002 35965208

[6] Ham WS Park JS Jang WS Kim J. Nephron-sparing approaches in upper tract urothelial carcinoma: current and future strategies. Biomedicines (2022) 10(9):2223. doi: 10.3390/biomedicines10092223 36140325 PMC9496458

[7] Musat MG Kwon CS Masters E Sikirica S Pijush DB Forsythe A. Treatment outcomes of high-risk non-muscle invasive bladder cancer (HR-NMIBC) in real-world evidence (RWE) studies: systematic literature review (SLR). Clinicoecon Outcomes Res (2022) 14:35–48. doi: 10.2147/CEOR.S341896 35046678 PMC8759992

[8] Klaassen Z Kamat AM Kassouf W Gontero P Villavicencio H Bellmunt J. Treatment strategy for newly diagnosed T1 high-grade bladder urothelial carcinoma: new insights and updated recommendations. Eur Urol (2018) 74(5):597–608. doi: 10.1016/j.eururo.2018.06.024 30017405

[9] Li R Metcalfe MJ Tabayoyong WB Guo CC Nogueras González GM Navai N. Using grade of recurrent tumor to guide further therapy while on bacillus calmette-guerin: low-grade recurrences are not benign. Eur Urol Oncol (2019) 2(3):286–93. doi: 10.1016/j.euo.2018.08.013 31200843

[10] Babjuk M Burger M Capoun O Cohen D Compérat EM Dominguez Escrig JL. European Association of urology guidelines on non-muscle-invasive bladder cancer (Ta, T1, and carcinoma in situ). Eur Urol (2022) 81(1):75–94. doi: 10.1016/j.eururo.2021.08.010 34511303

[11] van Rhijn BWG Hentschel AE Bründl J Compérat EM Hernández V Čapoun O. Prognostic value of the WHO1973 and WHO2004/2016 classification systems for grade in primary Ta/T1 non-muscle-invasive bladder cancer: a multicenter European association of urology non-muscle-invasive bladder cancer guidelines panel study. Eur Urol Oncol (2021) 4(2):182–91. doi: 10.1016/j.euo.2020.12.002 33423944

[12] Barrios W Abdollahi B Goyal M Song Q Suriawinata M Richards R. Bladder cancer prognosis using deep neural networks and histopathology images. J Pathol Inform (2022) 13:100135. doi: 10.1016/j.jpi.2022.100135 36268091 PMC9577122

[13] Zhang GM Sun H Shi B Jin ZY Xue HD. Quantitative CT texture analysis for evaluating histologic grade of urothelial carcinoma. Abdom Radiol (NY) (2017) 42(2):561–8. doi: 10.1007/s00261-016-0897-2 27604896

[14] Zhang X Xu X Tian Q Li B Wu Y Yang Z. Radiomics assessment of bladder cancer grade using texture features from diffusion-weighted imaging. J Magn Reson Imaging (2017) 46(5):1281–8. doi: 10.1002/jmri.25669 PMC5557707 28199039

[15] Wang H Hu D Yao H Chen M Li S Chen H. Radiomics analysis of multiparametric MRI for the preoperative evaluation of pathological grade in bladder cancer tumors. Eur Radiol (2019) 29(11):6182–90. doi: 10.1007/s00330-019-06222-8 31016445

[16] Zheng Z Xu F Gu Z Yan Y Xu T Liu S. Integrating multiparametric MRI radiomics features and the vesical imaging-reporting and data system (VI-RADS) for bladder cancer grading. Abdom Radiol (NY) (2021) 46(9):4311–23. doi: 10.1007/s00261-021-03108-6 33978825

[17] Lubner MG Smith AD Sandrasegaran K Sahani DV Pickhardt PJ. CT texture analysis: definitions, applications, biologic correlates, and challenges. Radiographics (2017) 37(5):1483–503. doi: 10.1148/rg.2017170056 28898189

[18] Gillies RJ Kinahan PE Hricak H. Radiomics: images are more than pictures, they are data. Radiology (2016) 278(2):563–77. doi: 10.1148/radiol.2015151169 PMC4734157 26579733

[19] Chang L Zhuang W Wu R Feng S Liu H Yu J. DARWIN: a highly flexible platform for imaging research in radiology. arXiv e-prints (2020). doi: 10.48550/arXiv.2009.00908

[20] Dong W Xiong S Lei P Wang X Liu H Liu Y. Application of a combined radiomics nomogram based on CE-CT in the preoperative prediction of thymomas risk categorization. Front Oncol (2022) 12:944005. doi: 10.3389/fonc.2022.944005 36081562 PMC9446086

[21] Hansel DE Amin MB Comperat E Cote RJ Knüchel R Montironi R. A contemporary update on pathology standards for bladder cancer: transurethral resection and radical cystectomy specimens. Eur Urol (2013) 63(2):321–32. doi: 10.1016/j.eururo.2012.10.008 23088996

[22] Zhang G Xu L Zhao L Mao L Li X Jin Z. CT-based radiomics to predict the pathological grade of bladder cancer. Eur Radiol (2020) 30(12):6749–56. doi: 10.1007/s00330-020-06893-8 32601949

[23] Feng C Zhou Z Huang Q Meng X Li Z Wang Y. Radiomics nomogram based on high-b-Value diffusion-weighted imaging for distinguishing the grade of bladder cancer. Life (Basel) (2022) 12(10):1510. doi: 10.3390/life12101510b 36294945 PMC9604764

[24] Lim ZF Ma PC. Emerging insights of tumor heterogeneity and drug resistance mechanisms in lung cancer targeted therapy. J Hematol Oncol (2019) 12(1):134. doi: 10.1186/s13045-019-0818-2 31815659 PMC6902404

[25] Wang F Zhang B Wu X Liu L Fang J Chen Q. Radiomic nomogram improves preoperative T category accuracy in locally advanced laryngeal carcinoma. Front Oncol (2019) 9:1064. doi: 10.3389/fonc.2019.01064 31681598 PMC6803547

[26] Li J Wu X Mao N Zheng G Zhang H Mou Y. Computed tomography-based radiomics model to predict central cervical lymph node metastases in papillary thyroid carcinoma: a multicenter study. Front Endocrinol (Lausanne) (2021) 12:741698. doi: 10.3389/fendo.2021.741698 34745008 PMC8567994

[27] Yang W Si Y Wang D Guo B. Automatic recognition of arrhythmia based on principal component analysis network and linear support vector machine. Comput Biol Med (2018) 101:22–32. doi: 10.1016/j.compbiomed.2018.08.003 30098452

[28] Shen C Liu Z Wang Z Guo J Zhang H Wang Y. Building CT radiomics based nomogram for preoperative esophageal cancer patients lymph node metastasis prediction. Transl Oncol (2018) 11(3):815–24. doi: 10.1016/j.tranon.2018.04.005 PMC6154864 29727831

[29] Chen S Jiang L Zheng X Shao J Wang T Zhang E. Clinical use of machine learning-based pathomics signature for diagnosis and survival prediction of bladder cancer. Cancer Sci (2021) 112(7):2905–14. doi: 10.1111/cas.14927 PMC8253293 33931925

[30] Çetinel G Mutlu F Gül S. Decision support system for breast lesions *via* dynamic contrast enhanced magnetic resonance imaging. Phys Eng Sci Med (2020) 43(3):1029–48. doi: 10.1007/s13246-020-00902-2 32691326

[31] Song H Jiao Y Wei W Ren X Shen C Qiu Z. Can pretreatment 18F-FDG PET tumor texture features predict the outcomes of osteosarcoma treated by neoadjuvant chemotherapy. Eur Radiol (2019) 29(7):3945–54. doi: 10.1007/s00330-019-06074-2 30859285

[32] Liu J Xu H Qing H Li Y Yang X He C. Comparison of radiomic models based on low-dose and standard-dose CT for prediction of adenocarcinomas and benign lesions in solid pulmonary nodules. Front Oncol (2020) 10:634298. doi: 10.3389/fonc.2020.634298 33604303 PMC7884759

[33] Pasini G Bini F Russo G Comelli A Marinozzi F Stefano A. matRadiomics: a novel and complete radiomics framework, from image visualization to predictive model. J Imaging (2022) 8(8):221. doi: 10.3390/jimaging8080221 36005464 PMC9410206

[34] Ferro M de Cobelli O Musi G Del Giudice F Carrieri G Busetto GM. Radiomics in prostate cancer: an up-to-date review. Ther Adv Urol (2022) 14:17562872221109020. doi: 10.1177/17562872221109020 35814914 PMC9260602

[35] Cacciamani GE Nassiri N Varghese B Maas M King KG Hwang D. Radiomics and bladder cancer: current status. Bladder Cancer (2020) 6:343. doi: 10.3233/BLC-200293

